# Reliability and Repeatability of the Instrument for the Assessment of Stress in Nursing Students (ASNS)

**DOI:** 10.3390/medicina55100634

**Published:** 2019-09-24

**Authors:** Marta Elena Losa-Iglesias, Raquel Jiménez-Fernández, Almudena Alameda-Cuesta, Maria Gema Cid-Exposito, Rocío Rodriguez-Vazquez, Ricardo Becerro-de-Bengoa-Vallejo

**Affiliations:** Facultad de Ciencias de la Salud, Universidad Rey Juan Carlos, Avenida de Atenas, 28040 Madrid, Spain; raquel.jimenez@urjc.es (R.J.-F.); almudena.alameda@urjc.es (A.A.-C.); gema.cid@urjc.es (M.G.C.-E.); rocio.vazquez@urjc.es (R.R.-V.); ribebeva@ucm.es (R.B.-d.-B.-V.)

**Keywords:** stress, nursing students, questionnaire

## Abstract

*Background and objectives*: Stress in nursing students is a very common experience, especially when they face clinical practice. The aims of this study were to perform a transcultural adaptation and to examine the reliability and repeatability of the Instrument for the Assessment of Stress in Nursing Students for a Spanish population. *Methods*: A test–retest analysis was carried out in two face-to-face sessions with the students with a lapse of 10 days between the two sessions. A cross-sectional descriptive study was carried out between the months of May and June 2018. Sixty-four nursing students were recruited with a consecutive sampling method that targeted individuals in the freshman class. *Results*: A good internal consistency was shown for the total score (α = 0.8861) and for each of the six domains. The test–retest reliability, using the Wilcoxon paired test, was not significant, indicating no differences between the total scores or the domain scores (*p* ≥ 0.05). Finally, Bland and Altman plots of visual distributions did not show differences between the total scores and the domain scores. *Conclusion*: The Instrument for the Assessment of Stress in Nursing Students was shown to be a reliable tool for measuring stress factors among Spanish nursing students.

## 1. Introduction

The presence of stress in students of all levels and ages is a reality in universities around the world [1]. The medical sciences have been reported as one of the branches of knowledge in which students experience high levels of stress [2,3].

Nursing students have a heavy load of theoretical and practical teaching hours with a high level of emotional demand [4]. This was demonstrated in several studies that found that stress among nursing students was very high, resulting in morbidity and psychological exhaustion [5,6].

There are not many validated measurement instruments to quantify stress in nursing students. One of those that is validated and that shows very good psychometric properties is the Instrument for the Assessment of Stress in Nursing Students (ASNS). The instrument is composed of 30 items grouped into six domains: performance of practical activities; professional communication; time management; environment; professional education; and theoretical activities. A factor analysis of the instrument’s psychometric properties confirmed that the conceptual model was acceptable, resulting in Cronbach’s alphas ranging from 0.71 to 0.87 [7].

This instrument has been used in previous studies, and the results were very useful for assessing the stress factors among nursing students, which led to good results [8,9,10,11].

The ASNS had not been adapted to the Spanish language or validated in a Spanish population. Therefore, this study aimed to perform a transcultural adaptation and test the reliability and repeatability of the Instrument for the Assessment of Stress in Nursing Students in a Spanish population.

## 2. Materials and Methods

### 2.1. Study Design

A cross-sectional descriptive study was carried out between the months of May and June 2018. Transcultural adaptation and reliability and a repeatability assessment were carried out using the ASNS with nursing students who were recruited with a consecutive sampling method from the freshman class at the University of Rey Juan Carlos (URJC) (Madrid, Spain). The ages of the participants ranged from 19 to 33 years old. The exclusion criteria were prior history working in medical settings, such as a nursing assistant position or any kind of work at a hospital, day care center, or similar institution; refusal to sign the informed consent form; inability to understand the instructions for completing the test; and being of a nationality other than Spanish [7] .

This research was approved by the Ethics Committee of the Hospital Universitario Clínico San Carlos (Madrid, Spain), number 18/278-E, on 6 June 2018.

### 2.2. Translation Procedure

The internationally recommended forward/reverse translation protocol was applied for the translation, transcultural adaptation, and validation of the constructs [12,13]. Finally, the proofread version of the ASNS was composed of the same items (30 items) and Likert scale that were used in the original version.

### 2.3. Test–Retest Reliability and Sample Size

Test–retest was carried out during two face-to-face sessions with the students with a lapse of 10 days between the two sessions. In addition to the ASNS items, socio-demographic data such as age and gender were requested in the session. Participants were recruited from the Faculty of Health Sciences of URJC, Spain. Considering the correlation with a CCI of 0.40 and a 95% confidence interval (CI) for a two-tailed test, an α error of 0.05, and a desired analysis power of 80% (error β = 20%), the final sample included at least 53 participants.

### 2.4. Statistical Analysis

Independent Student *t*-tests were performed to determine whether there were statistically significant differences when a normal distribution was shown. Considering the total score and each domain score, internal consistency and reliability were analyzed using the intraclass correlation coefficient (ICC) and Cronbach’s alpha (α) with a confidence interval of 95% (95% CI). For the statistical analysis, we used a bidirectional random effects model (2.1), single measures, absolute agreement, and ICC to express reliability. In addition, a Wilcoxon paired test was applied to test the systematic differences between the original test and the new test. A Bland and Altman plot was also analyzed to assess agreement and heteroscedasticity [14] .

## 3. Results

### 3.1. Translation

The final sample that completed the two questionnaires comprised of 64 individuals: 56 women and 8 men. The age of the students ranged from 18 to 33 with a mean age of 19 ± 2.18 years old. The forward translations were performed with no discrepancies between the two versions.

### 3.2. Reliability and Repeatability

The Cronbach’s alpha for the total score shows a good internal consistency in the post-test, α = 0.8861 with a 95% lower confidence limit of 0.8467; the ICC with a 95% confidence interval was 0.5162 (0.2036–0.7061). Additionally, we found a very good internal consistency in all the domains of the questionnaire: the performance of practical activities had a Cronbach’s alpha of 0.8602; professional communication, α = 0.8785; time management, α = 0.876; environment, α = 0.9063; professional education, α = 0.864; and finally, theoretical activities, α = 0.8646.

Table 1 shows the results of the concordance of the test–retest questionnaire by the 30 items and 6 domains.

The test-retest reliability, using the Wilcoxon paired test, was not significant (ICC 95%), indicating that there were no systematic differences in the total scores of the questionnaires (*p* > 0.05). For the domains scores, statistically significant differences were not found in the mean (SD) difference between test and retest for any of the domains.

**Figure 1:**
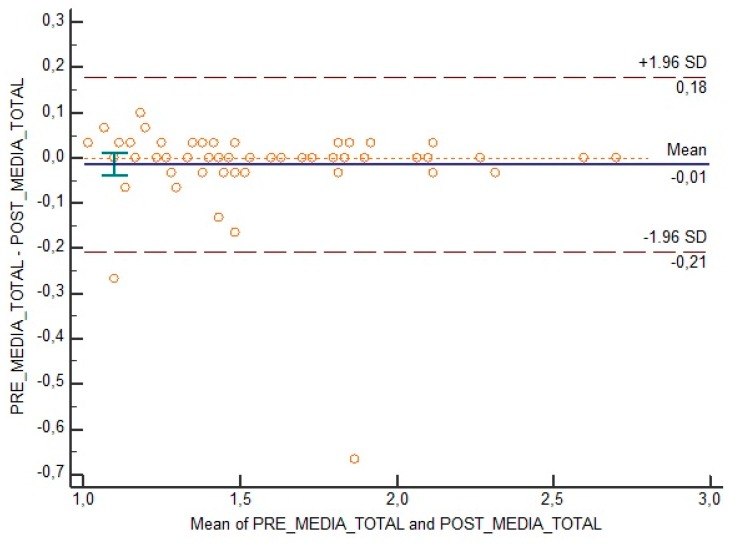
Bland and Altman plot for total items of adapted ASNS.

The 95% limit of agreements and Bland and Altman plots did not show changes or relevant differences from test to retest for the total scores (Figure 1) or for the six different domains of the questionnaire.

## 4. Discussion

According to Wild [13], the guidelines for the process of cross-cultural adaptation of self-reported measures, and by following George and Mallery’s [15] recommendations to evaluate Cronbach’s alpha coefficients, the adapted ASNS for a Spanish population is a reliable and valid questionnaire for measuring stress in nursing students. Our results are comparable to and slightly better than those obtained with the original validation of the questionnaire by Costa & Polak [7] , as we found higher Cronbach’s alpha values for the total test scores and for all the domains and better ICC values by domain and item. This could be because our sample was similar to the original validation sample on age and sex variables, though our sample came from exclusively freshman students with no previous experience in clinical settings, and this aspect can have a positive influence since there is no sample contamination or bias [16] .

The Spanish version of the ASNS was shown to be a valid and reliable tool with very good internal consistency for evaluating stress in nursing students due to performance of practical activities (0.8602), professional communication (0.8785), time management (0.876), environment (0.9063), professional education (0.864), and theoretical activities (0.8646). The questionnaire shows excellent reproducibility; all the *p* values from the Wilcoxon paired test were not statistically significant.

In general, the Bland and Altman method shows whether there are differences between the tests (the pre- and post-test) to check the reproducibility of the results of the test and whether the differences are systematic or random.

Comparing our reliability data (α = 0.8861) with those of other tools used to measure nursing stress in freshman students, we found results similar to those of Sheu et al. [17] with an α of 0.89, Shaban et al. [18] with an α of 0.87, and Karabacak et al. [19] with an α of 0.70.

The validation of a tool to measure stress in nursing students in Spain was also performed by Zupiria et al. [20], and these authors reported that the KEZKAK questionnaire had an α of 0.95. This value is higher than that of our questionnaire. In this study, the researchers included nursing students in all years of study, so this value cannot be compared with our value. The value obtained by Zupiria et al. [20] is in accordance with that obtained in another study with a heterogeneous sample conducted by Chen and Hung [21] which obtained an α of 0.91. Finally, age and sex were not considered in this validation due to the sample size.

## 5. Conclusions

The Instrument for the Assessment of Stress in Nursing Students (ASNS) for measuring stress factors among nursing students was shown to be a reliable tool for use with the Spanish students and may be used to obtain total scores or domain scores in further studies.

## Figures and Tables

**Table 1:** Analysis of intraclass correlation coefficient (ICC) between pre- and post-test of the adapted Assessment of Stress in Nursing Students (ASNS).

Items/Domains	ICC
**Domain: Performance of practical activities**	0.984 (0.973–0.990)
Item 5: New situations one may experience in clinical practice	0.941 (0.903–0.964)
Item 7: Environment of the training clinical ward	0.949 (0.916–0.969)
Item 9: Fear of making mistakes while assisting patients	0.988 (0.981–0.993)
Item 21: Feeling of not having enough knowledge for the practical test	0.996 (0.993–0.997)
Item 4: Performing the general assistance procedures	1 (1–1)
Item 12: Performing certain assistance procedures	1 (1–1)
**Domain: Professional communication**	0.933 (0.890–0.959)
Item 6: Communication with the other professionals at the training ward	0.959 (0.932–0.975)
Item 8: Communication with professionals of other sectors at the training ward	0.963 (0.939–0.977)
Item 16: Perception of difficulties regarding relationships with other nursing professionals	0.958 (0.931–0.975)
Item 20: Identification of contradictory attitudes in the other professionals	966 (0.944–0.979)
**Domain: Time management**	0.989 (0.982–0.993)
Item 18: Little time to spend with family members	0.988 (0.980–0.993)
Item 3: Reduced social interactions because of feelings of loneliness	1 (1–1)
Item 26: Lack of leisure time	0.987 (0.978–0.992)
Item 30: Lack of time to rest	0.968 (0.946–0.980)
Item 23: Time demanded by the professor to prepare extra class activities	0.996 (0.994–0.998)
**Domain: Environment**	0.989 (0.982–0.993)
Item 11: Distance between school and residence	0.969 (0.949–0.981)
Item 29: Public transportation used to go to the training place	0.984 (0.974–0.990)
Item 22: Public transportation used to go to school	0.994 (0.991–0.997)
Item 24: Distance between most training places and residences	0.978 (0.964–0.987)
**Domain: Professional education**	0.995 (0.993–0.997)
Item 1: Concerns about professional future	0.965 (0.974–0.990)
Item 15: Similarities between situations experienced during the training process and those that may be experienced during professional life	1 (1–1)
Item 17: Thinking of situations that may be experienced during professional life	0.978 (0.964–0.987)
Item 19: Perceptions of professional responsibility while doing the training program	0.971 (0.952–0.982)
Item 25: Experiencing activities in the training field as a nursing student	1 (1–1)
Item 27: Perceptions of the theoretical knowledge acquired during the course	0.984 (0.973–0.990)
**Domain: Theoretical activities**	0.993 (0.988–0.995)
Item 10: Format of method used to assess theoretical content	0.988 (0.980–0.993)
Item 13: Feelings of insecurity or fear while taking theoretical exams	0.994 (0.990–0.996)
Item 14: Level of difficulty of extra class assignments	0.995 (0.991–0.997)
Item 2: Obligation to do extra class assignments	0.988 (0.981–0.993)
Item 28: Understanding the theoretical and practical content taught in the classroom	0.982 (0.971–0.989)
